# Mitochondrial stress protein HSP60 regulates ER stress-induced hepatic lipogenesis

**DOI:** 10.1530/JME-19-0207

**Published:** 2019-12-04

**Authors:** Ting Xiao, Xiuci Liang, Hailan Liu, Feng Zhang, Wen Meng, Fang Hu

**Affiliations:** 1 Department of Metabolism and Endocrinology, The Second Xiangya Hospital, Central South University, Changsha, Hunan, China; 2 National Clinical Research Center for Metabolic Diseases, Changsha, Hunan, China; 3 Metabolic Syndrome Research Center, Central South University, Changsha, Hunan, China

**Keywords:** HSP60, ER stress, mitochondrial stress, mTORC1-SREBP1 signaling, hepatic lipogenesis

## Abstract

Endoplasmic reticulum (ER) stress and mitochondrial dysfunction are associated with hepatic steatosis and insulin resistance. Molecular mechanisms underlying ER stress and/or mitochondrial dysfunction that cause metabolic disorders and hepatic steatosis remain to be fully understood. Here, we found that a high fat diet (HFD) or chemically induced ER stress can stimulate mitochondrial stress protein HSP60 expression, impair mitochondrial respiration, and decrease mitochondrial membrane potential in mouse hepatocytes. HSP60 overexpression promotes ER stress and hepatic lipogenic protein expression and impairs insulin signaling in mouse hepatocytes. Mechanistically, HSP60 regulates ER stress-induced hepatic lipogenesis via the mTORC1-SREBP1 signaling pathway. These results suggest that HSP60 is an important ER and mitochondrial stress cross-talking protein and may control ER stress-induced hepatic lipogenesis and insulin resistance.

## Introduction

Endoplasmic reticulum (ER) stress due to high fat diet (HFD) feeding or genetic obesity is associated with hepatic steatosis and insulin resistance (Kammoun *et al*. 2009, Yoshiuchi *et al*. 2009, Ye *et al*. 2010, Meng *et al*. 2017, Luo *et al*. 2018). Hepatic steatosis is characterized by an excessive accumulation of lipids that ultimately leads to disruption of tissue architecture and organ dysfunction. However, the precise mechanisms by which ER stress causes metabolic disorders and hepatic steatosis are yet to be fully elucidated. Recent studies have revealed that hepatic mTORC1-SREBP1 (sterol regulatory element-binding protein 1c) signaling plays a key role in regulating systemic hepatic lipid metabolism (Porstmann *et al*. 2008, Düvel *et al*. 2010, Yecies *et al*. 2011, Owen *et al*. 2012, Cai *et al*. 2016). Not only does mTORC1 regulate Srebp-1 gene transcription (Bakan & Laplante 2012) but it also promotes the transformation of primordial SREBP (p-SREBP) to functional mature SREBP (m-SREBP) (Han & Wang 2018). However, the mechanisms underlying ER stress-induced hepatic lipogenesis and steatosis via the mTORC1- SREBP1 signaling pathway are yet to be completely elucidated.

Similar to the stress response in the ER, mitochondrial stress is characterized by a mitochondrial unfolded protein response (UPR^mt^) and initiation of a retrograde stress signaling pathway accompanied by impaired mitochondrial function and membrane potential (Haynes *et al*. 2007, Broadley & Hartl 2008, Haynes & Ron 2010, Hu & Liu 2011). HSP60, an important mitochondrial stress protein, has been shown to play key roles in the protein synthesis, folding, and delivery of misfolded proteins to proteolytic enzymes in the mitochondrial matrix (Tatsuta 2009, Haynes & Ron 2010, Hu & Liu 2011, Marino Gammazza *et al*. 2018). Recent studies have showed that mitochondrial stress is associated with mitochondrial dysfunction and metabolic disorders such as hepatic steatosis, insulin resistance, and type 2 diabetes (Venojarvi *et al*. 2008, Hu & Liu 2011, Comert *et al*. 2019, Einer *et al*. 2019). ER and mitochondria have partially overlapping functions, and there is strong crosstalk between the two organelles to regulate hepatic steatosis (Haynes & Ron 2010, Hu & Liu 2011, Ghemrawi *et al*. 2018). However, the cause-and-effect relationship between ER and mitochondrial stress in response to nutrients overloading are yet to be clearly elucidated.

In current studies, we showed that HFD induced both ER stress and mitochondrial stress in the liver of C57/B6 mice. In addition, we found that ER stress also induced mitochondrial stress protein HSP60 expression and impaired mitochondrial function and membrane potential in mouse hepatocytes. The overexpression of HSP60 enhanced ER stress and impaired insulin signaling. Importantly, our studies demonstrate HSP60-regulated ER stress-induced hepatic lipogenesis via the mTORC1-SREBP1 signaling pathway. Thus, HFD and ER stress-induced HSP60 might be an important ER and mitochondrial stress cross-talking protein that plays a key role in the regulation of hepatic lipid metabolism.

## Materials and methods

### Chemicals and antibodies

ER stress-inducer tunicamycin (TM) and stress blocker tauroursodeoxycholic acid (TUDCA) were purchased from Sigma. The primary antibodies against mouse β-actin, phospho-Akt (Ser473), AKT, CHOP, phospho-FOXO1(FOXO1-P), FOXO1, mTORC1, p70S6K, and phospho-p70S6K (S6K-P) were obtained from Cell Signaling Technology Inc. The primary antibodies against HSP60, GRP78, SREBP1, fatty acid synthase (FAS), ACC, and phospho-ACC (ACC-P) were obtained from Santa Cruz Biotechnology. The horseradish peroxidase-conjugated secondary antibodies were obtained from Promega.

### Cell culture, treatment, and transfection

HepIR mouse liver cells were cultured in MEMα (Gibco) culture medium supplemented with 4% fetal bovine serum (FBS; Gibco) and 1% penicillin/streptomycin (Gibco) in 37°C and 5% CO_2_ incubator. ER stress was induced by treating cells with tunicamycin (TM) (0.1 nM) for 24 h. For HSP60 overexpression, HepIR cells were infected with control or HSP60 expressing vector by lentivirus for 8 h, and then washed and incubated for another 48 h before collection. To knock down endogenous *Hsp60* gene expression, HepIR cells were transfected with *Hsp60*-siRNA (forward: 5′-GGAAGUCCCA AAGUAA CAATT-3′; reverse: 5′-UUGUUACUUUGGGACUUCCTT-3′) or control NC-siRNA (forward: 5′-UUCUCCGAACGUGUCACGUTT-3′; reverse 5′-ACGUGACACGUUCGGAGATT-3′) using Lipofectamine 2000 (Invitrogen). Cells were collected after 48 h transfection.

### Animal studies

Six-week-old male mice (C57BL/6J) were purchased from Slac Laboratory Animal Inc. (Shanghai, China) and housed in a temperature-controlled environment with a 12:12 h light/dark cycle. The mice had free access to food and water *ad libitum*. After a one week adaptive period, the mice were randomly divided into two weight-matched groups and fed with either a high fat diet (HFD) (60% kcal; #12492) or a control diet (10% kcal; #12540B) (Research Diets, Inc., New Brunswick, USA). After 8 weeks of feeding, the animals were killed and their livers were rapidly isolated, followed by immediate freezing in liquid nitrogen and storing at −80°C before analyses. ER stress was induced by i.p. administration of TM (Sigma). Briefly, a single i.p. injection of TM (1 mg/kg) or equal volumes of vehicle (saline) were applied to the male mice, and 16 h after injection the mice were killed and the tissues were collected. All protocols for animal use and maintenance were approved by the Central South University Animal Care and Use Committee.

### Western blots

For protein extraction, approximately 30 mg of frozen tissue was homogenized in 400 µl RIPA buffer (Beyotime Institute of Biotechnology, Shanghai, China). Extracts were spun down and the fat layer and cell debris were removed. Protein concentration was determined by the BCA kit. Equal amounts of proteins from each sample were loaded and separated by SDS-PAGE. Proteins were transferred to polyvinylidene difluoride membrane and incubated with a blocking buffer (5% BSA in 20 mM Tris-HCl, pH 7.5, 137 mM NaCl, and 0.1% Tween 20) for 1 h at room temperature and then incubated with primary antibodies at 4°C overnight. The membrane was incubated with secondary antibodies (1:5000 to 1:10,000 dilution) for 1 h at room temperature and detected with enhanced chemiluminescence (Bio-Rad).

### Mitochondrial membrane potential measurement

Mitochondrial membrane potential (∆ψm) was measured using the fluorescent probe JC-1 (5,5′,6,6′-tetrachloro-1,1′,3,3′-tetraethyl-benzimidazol carbocyanine iodide) (Invitrogen) as described previously (Woollacott & Simpson 2001). JC-1 is a cationic dye that is accumulated in mitochondria following membrane potential. Briefly, after treatment with or without 0.1 μg/mL TM for 24 h, cells were trypsinised and resuspended with Krebs-Ringer HEPES buffer (120 mM NaCl, 1.9 mM CaCl_2_, 4.6 mM KCl, 25 mM HEPES, 1 mM MgSO_4_, 1.2 mM KH_2_PO_4_, 1% (w/v) BSA, pH 7.4). Then 1 × 10^6^ cells were incubated with 1 µg/ml JC1 at 37°C for 20 min, immediately followed by the measuring of red (excitation 488 nm, emission 575 nm) to green fluorescence (excitation 488 nm, emission 530 nm) by a flow cytometry system (BD Biosciences, USA). Mitochondrial depolarization was achieved by treating cells with carbonyl cyanide 4-(trifluoromethoxy) phenylhydrazone (FCCP; 10 µM) (20 min, 37°C), an uncoupling agent that abolishes the ∆ψm.

### Oxygen consumption

Mitochondrial oxygen consumption in intact cells were measured with the Seahorse Bioscience XF-24 analyzer (Seahorse Bioscience, Billerica, MA, USA) and reported as oxygen consumption rate (OCR) as described previously (Brand & Nicholls 2011). HepIR cells were seeded at 15 000 cells/well into XF-24 culture microplates and cultured overnight at 37°C with 5% CO_2_. During the 24 h post-treatment with or without TM, the medium was replaced with pre-warmed 600 μl of sodium carbonate-free DMEM for 1 h. Each experimental condition was analyzed using four to six biological replicates. The following reagents including 1 μM oligomycin (Sigma-Aldrich), 1 μM FCCP (Sigma-Aldrich) or 1 μM rotenone (Sigma-Aldrich) were added to block state III respiration, induce uncoupling, or shut down mitochondrial respiration, respectively. Data were normalized to protein content.

### Oil red O staining

Cultured cells were fixed with 4% paraformaldehyde for 30 min and washed with PBS. The fixed cells were stained with 1% Oil red O for 1 h at room temperature and rinsed with PBS thrice. Oil red O-stained lipid-laden cells in different experimental groups were observed with an Olympus microscope.

### Statistical analysis

All data were reported as average ± s.e.m. Differences between control and treatment were analyzed by unpaired, two-tailed Student’s *t*-test, and statistical significance was set as *P* < 0.05.

## Results

### ER stress-induced mitochondrial stress protein HSP60 expression in liver of mice

**Figure 1:**
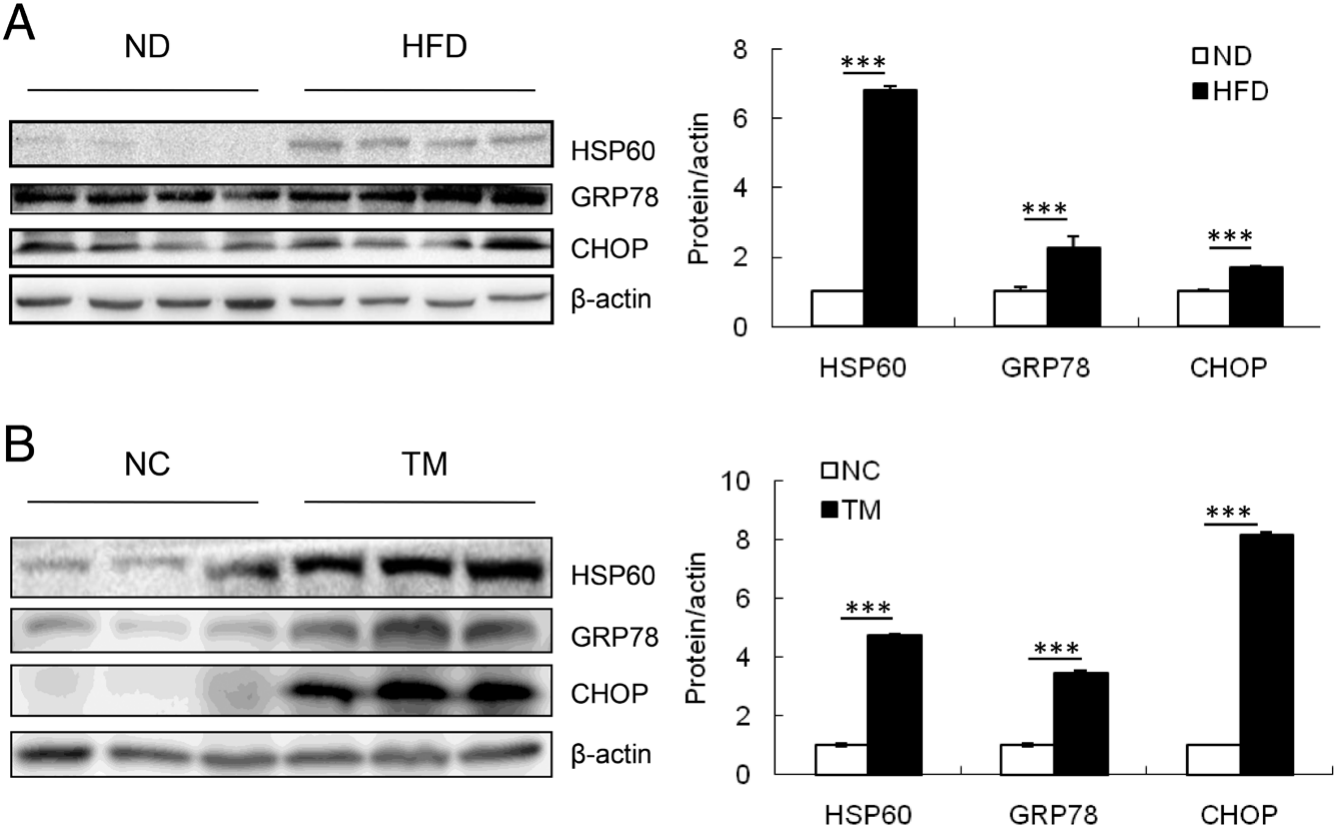
ER stress-induced mitochondrial stress protein HSP60 expression in liver of mice. (A) Western blot analyses of the protein levels of HSP60, GRP78, and CHOP in liver of mice after a 8-week HFD or ND feeding regimen starting at 8 weeks of age. (B) The protein levels of HSP60, GRP78, and CHOP in liver of mice after i.p. injection of 1 mg/kg TM for 16 h. Data are presented as mean ± s.e.m. ***P < 0.001. HFD: high fat diet; ND: normal diet; TM: tunicamycin.

Recent studies have revealed that ER stress and mitochondrial dysfunction were induced in the liver of HFD-fed mice accompanied by hepatic lipogenesis and steatosis (Hu & Liu 2011, Yecies *et al*. 2011, Ghemrawi *et al*. 2018, Luo *et al*. 2018). While ER and mitochondria are subjected to distinct regulations in response to stress, these two organelles are connected at multiple levels during the stress response (Hu & Liu 2011). As the mechanism of HFD and ER stress-induced-hepatic lipogenesis is still poorly understood, we sought to determine whether mitochondrial stress contributes to lipid accumulation in the liver of mice. As expected, HFD induced the high expression of GRP78 and C/EBP-homologous protein (CHOP), which are ER stress signaling marker proteins (Fig. 1A). Interestingly, we found that HSP60, an important marker of mitochondrial stress, was also greatly increased in the liver of HFD-fed mice (Fig. 1A), suggesting that HFD induces ER stress as well as mitochondrial stress in the liver of mice.

In order to determine whether ER stress induced mitochondrial stress *in vivo*, we injected TM, an inhibitor of the ER-specific calcium ATPase, to induce ER stress in C57/B6 mice. As expected, the expression levels of GRP78 and CHOP were significantly increased. Meanwhile, the expression of mitochondrial stress protein HSP60 in the liver of TM-injected mice also increased significantly (Fig. 1B). Thus, the results show that both HFD and ER stress induced HSP60 expression in the liver of C57/B6 mice.

### ER stress impaired mitochondrial function and membrane potential in mouse hepatocytes

**Figure 2:**
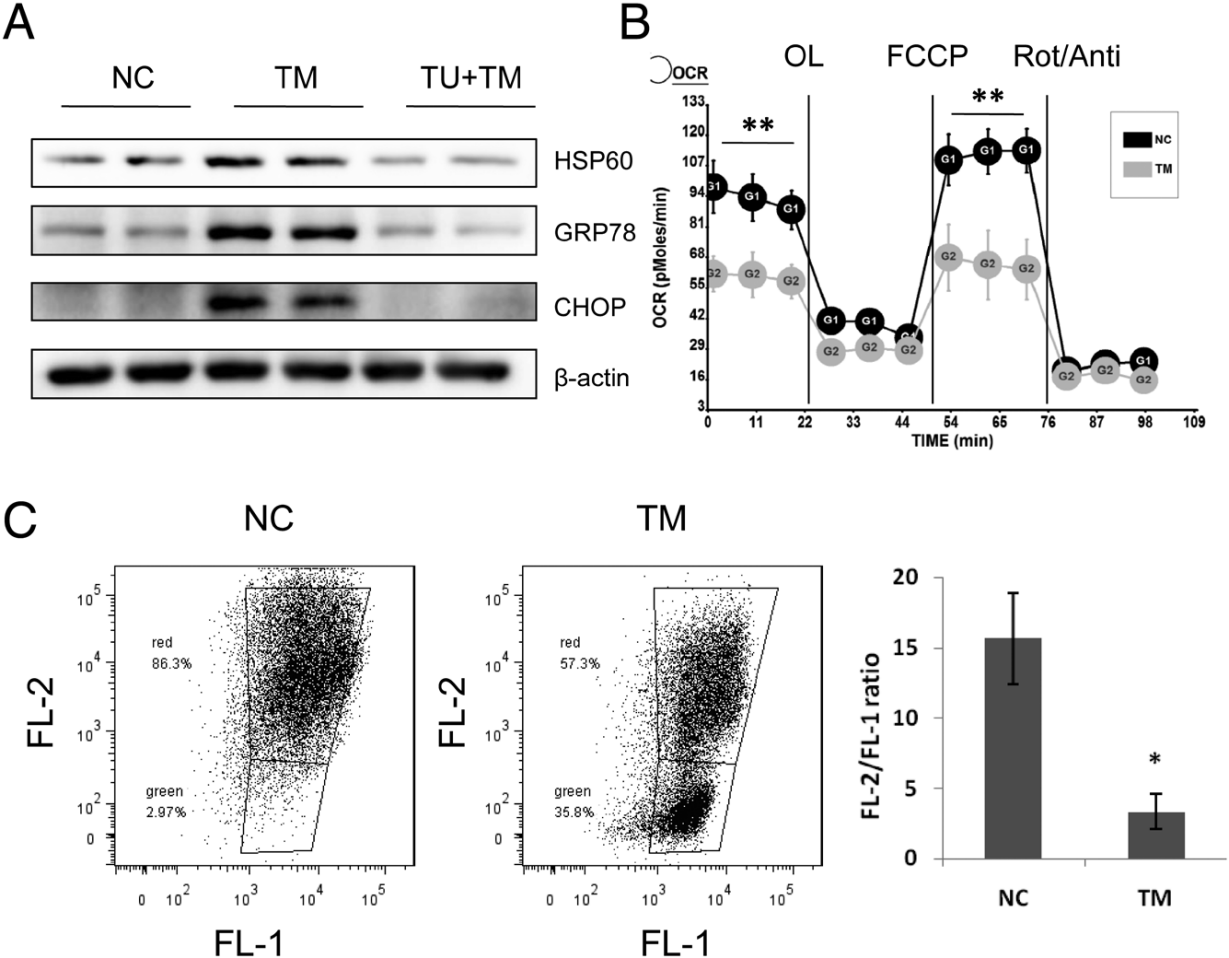
ER stress impaired mitochondrial function and membrane potential in mouse hepatocytes. (A) Western blot analyses of the protein levels of HSP60, GRP78, and CHOP in HepIR cells treated with or without TM (0.1 nM) for 24 h, followed by treatment with or without TUDCA (100 μM) for 1 h. (B) OCR of HepIR cells treated by TM. VO_2_ consumption was normalized to protein content. (C) Flow cytometry analysis for mitochondrial membrane potential in HepIR cells treated with or without TM. Data are presented as mean ± s.e.m. *P < 0.05; **P < 0.01. OCR: oxygen consumption rate; OL: oligomycin; Rot: rotenone; Anti: antimycin; TM: tunicamycin; TUDCA: tauroursodeoxycholic acid.

To further determine whether ER stress directly induces mitochondrial dysfunction in a cellular autonomous manner, we used TM to induce ER stress and examined the expression levels of HSP60 in HepIR cells. As expected, the use of TM led to a significant increase in the level of ER stress in hepatocytes, which was demonstrated by increased CHOP and GRP78 expression levels (Fig. 2A). Concurrent with the increased level of ER stress, the expression level of HSP60 also increased in the TM-treated cells compared with that in the control HepIR cells, while the use of ER stress-reducing chemical chaperone TUDCA caused a decline in TM-induced ER stress protein (CHOP and GRP78) and mitochondrial stress protein HSP60 expression (Fig. 2A). Therefore, the results show that ER stress directly induced mitochondrial stress in mouse hepatocytes.

To determine whether ER stress alters mitochondrial respiration, we measured the oxygen consumption in HepIR cells that were treated either with or without TM. Through TM treatment, the basal and FCCP-stimulated maximal respiration was significantly suppressed in HepIR cells (Fig. 2B). JC-1 is a cationic fluorescent dye that is accumulated in mitochondria in a potential-dependent manner. A gradual increase in JC-1 green fluorescence and loss of orange fluorescence represents cells with compromised mitochondrial membrane potential. We then went on to measure the effect of ER stress on mitochondrial membrane potential in HepIR cells and found that the JC-1 green fluorescence increased significantly in HepIR cells treated with TM, and the percentage of such cells increased from 2.97% to 35.8% (Fig. 2C). The mitochondrial transmembrane potential (indicated by FL-2/FL-1 ratio) was decreased in early apoptotic cells by the ER stress inducer TM (Fig. 2C). Thus, these results suggest that ER stress impaired mitochondrial respiration and membrane potential.

### HSP60 overexpression induced ER stress and impaired insulin signaling in mouse hepatocytes

**Figure 3:**
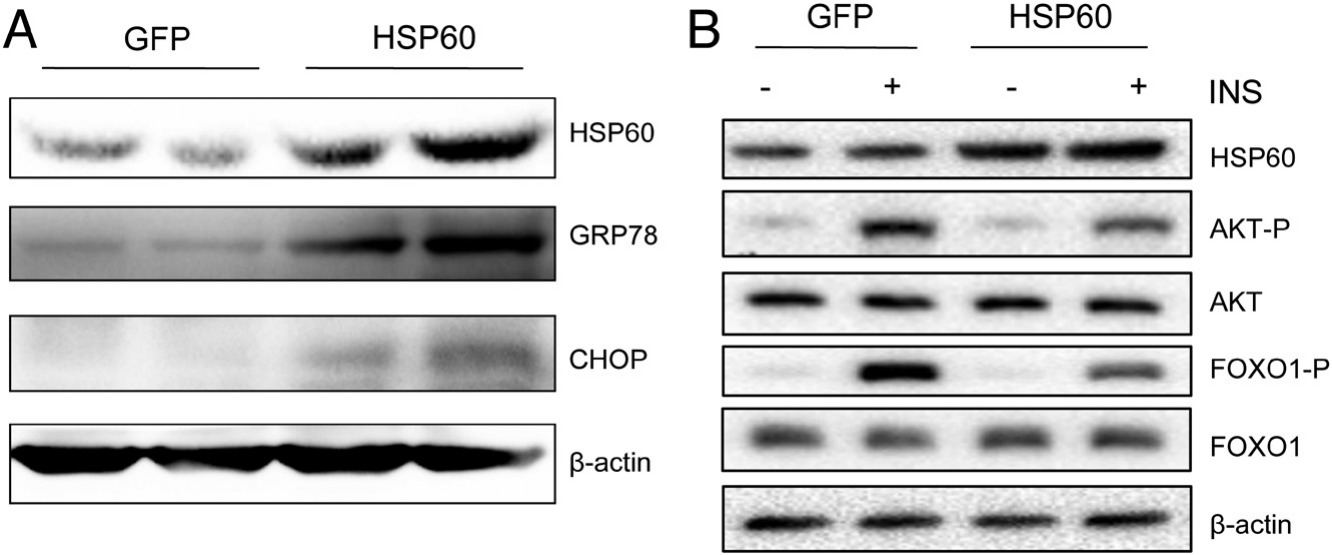
HSP60 overexpression induced ER stress and impaired insulin signaling in mouse hepatocytes. (A) Western blot analyses of the protein levels of HSP60, GRP78, and CHOP in mouse hepatocytes overexpressing HSP60. (B) Mouse hepatocytes were infected with lentivirus encoding HSP60, followed by treatment with or without insulin (10 nM) for 10 min, and expressions of the indicated proteins were analyzed by Western blot.

While numerous studies have demonstrated that a close biochemical and physiological connection exists between ER and mitochondria (Franzini-Armstrong 2007, Giorgi *et al*. 2009, Beller *et al*. 2010), an interesting question that remains unanswered is the cause-and-effect relationship between ER and mitochondrial stress. Since our studies showed that ER stress induced mitochondrial stress, we then went on to determine whether mitochondrial stress can induce ER stress. Overexpression of HSP60 stimulated ER stress levels in the hepatocytes of mice greatly, as demonstrated by increased CHOP and GRP78 expression levels (Fig. 3A). It was reported that insulin sensitivity was regulated by cross-talking between mitochondria and ER during stress (Hu & Liu 2011, Ghemrawi *et al*. 2018). To decide whether overexpression of HSP60 regulates insulin sensitivity, we treated HSP60-overexpressed hepatocytes with or without insulin (Fig. 3B). We found that insulin stimulated the phosphorylation of AKT and FOXO1, and this effect was impaired greatly by overexpressing HSP60 in HepIR cells (Fig. 3B), indicating that HSP60 overexpression impaired insulin signaling and insulin sensitivity in mouse hepatocytes.

### ER stress induced hepatic lipogenesis via HSP60-mediated mTORC1-SREBP1 signaling pathway

**Figure 4:**
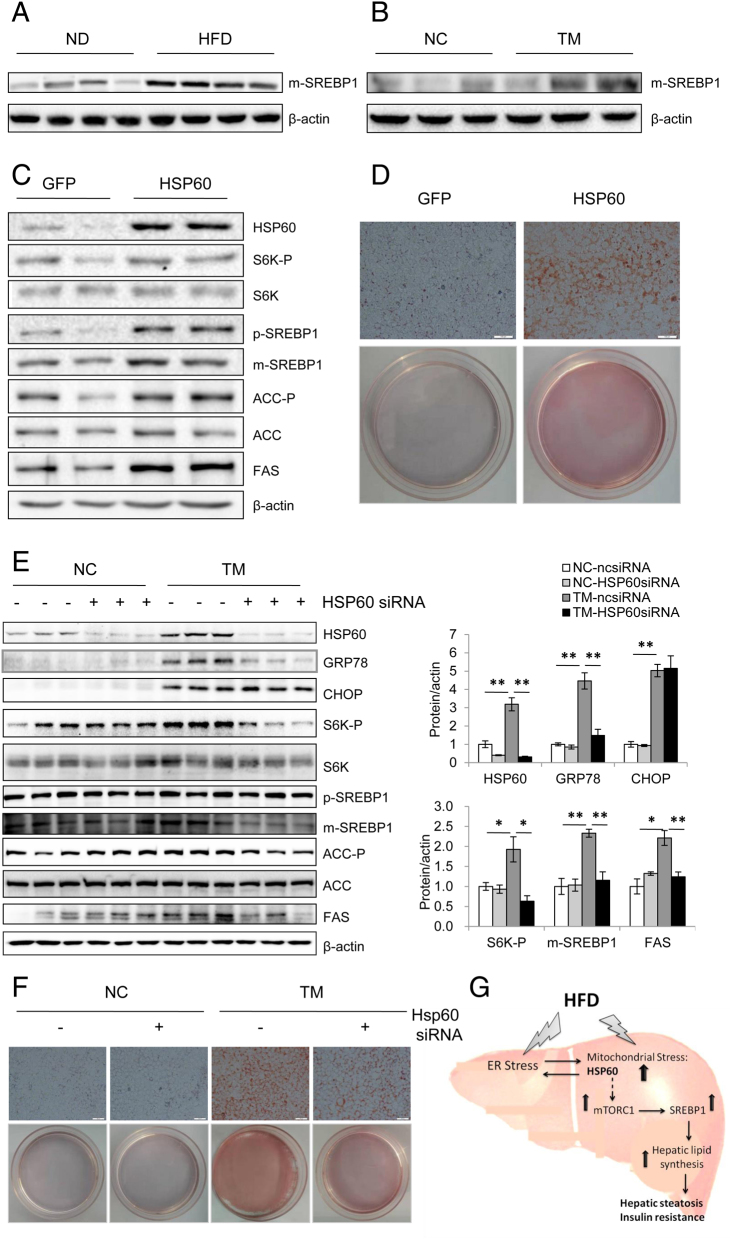
ER stress induced hepatic lipogenesis via HSP60-mediated mTORC1-SREBP1 signaling pathway. (A) Western blot analyses of the protein level of SREBP1 in liver of mice after a 8-week HFD or ND feeding regimen starting at 8 weeks of age. (B) The protein level of SREBP1 in liver of mice after i.p. injection of 1 mg/kg TM for 16 h. (C) Western blot analyses of the phosphorylation and protein levels of S6K, ACC, FAS, and primordial SREBP (p-SREBP) and mature SREBP (m-SREBP) in mouse hepatocytes overexpressing HSP60. (D) Oil red O staining in mouse hepatocytes overexpressing HSP60 (Scale bar = 100 μm). (E) Mouse hepatocytes were treated with *Hsp60* siRNA or their control siRNA, followed by treatment with or without TM (0.1 nM) for 24 h, and expressions of the indicated proteins were analyzed by Western blot. (F) Mouse hepatocytes were treated with *Hsp60* siRNA or their control siRNA, followed by treatment with or without TM (0.1 nM) for 24 h and stained with Oil red O (Scale bar = 100 μm). (G) A proposed model showing the role of HSP60 in HFD and ER stress-induced hepatic steatosis and insulin resistance. The HFD and ER stress-induced mitochondrial stress protein HSP60 promotes lipid accumulation via mTORC1-SREBP1 signaling pathway, leading to hepatic steatosis and insulin resistance. The dashed arrow indicates direct or indirect action. A full colour version of this figure is available at https://doi.org/10.1530/JME-19-0207.

As depicted in Fig. 1A and B, HFD and TM induced both ER and mitochondrial stress in the liver of mice. To determine whether ER stress-stimulated mitochondrial stress affects hepatic lipid metabolism, we measured the level of SREBP1 protein, a key transcription factor in promoting hepatic lipogenesis. We found that SREBP1 levels were increased in the liver of HFD-fed mice (Fig. 4A) and TM-treated mice (Fig. 4B). Interestingly, in agreement with these findings, overexpression of HSP60 stimulated the hepatic SREBP1, FAS experssion (Fig. 4C), as well as the mTORC1 signaling in mouse hepatocytes. Furthermore, Oil red O staining experiments showed that overexpression of HSP60 promoted hepatic lipogenesis (Fig. 4D).

To further define the role of HSP60 in the regulation of hepatic lipid metabolism, we knocked out HSP60 under the action of TM in mouse hepatocytes. We found that treating mouse hepatocytes with TM led to an increase in the expression of HSP60, and a marked increase in mTOR signaling and SREBP1 associated lipogenesis, as demonstrated by enhanced S6K-P and FAS, and increased m-SREBP1 expression, while the knockdown of HSP60 suppressed these protein levels, indicating inactivation of the mTORC1-SREBP1 signaling pathway (Fig. 4E). On the other hand, the knockdown of HSP60 blocked ER stress-induced hepatic lipogenesis as determined by Oil red O staining (Fig. 4F). Together, these results suggest that hepatic HSP60 could be a critical effector to the downstream of ER stress for the induction of mTORC1-SREBP1 signaling and maybe involved in mTORC1-SREBP1-regulated hepatic lipid metabolism.

## Discussion

In this study, we have confirmed that ER stress is important in the regulation of hepatic lipogenesis, which was consistent with the previous reports about ER stress-induced hepatic steatosis and insulin resistance (Hu & Liu 2011, Yecies *et al*. 2011, Chen *et al*. 2017, Ghemrawi *et al*. 2018, Han & Wang 2018, Luo *et al*. 2018). More importantly, we have also shown that ER stress induced mitochondrial stress protein HSP60 expression and impaired mitochondrial function in mouse hepatocytes (Fig. 2A and C). HSP60 overexpression deteriorated ER stress and impaired insulin signaling (Fig. 3A and B). Furthermore, we have demonstrated that the knockdown of HSP60 inhibited ER stress-induced mTORC1-SREBP1 signaling pathway is essential in regulating hepatic lipid metabolism (Fig. 4E and F). We first identified ER stress-induced mitochondrial stress protein as the potential mediator of mTORC1-SREBP1 signaling in hepatic lipid metabolism. Our results revealed a previously unrecognized mechanism, in which HFD and ER stress induced hepatic steatosis and insulin resistance.

Previous studies have shown that mitochondrial stress caused by obesity and/or metabolic dysfunction induces the upregulation of mitochondrial stress protein and accumulation of ROS to disrupt membrane potential and uncoupling of OXPHOS, leading to decreased mitochondrial respiration and mitochondrial dysfunction (Biswas *et al*. 1999, Amuthan *et al*. 2002, Haynes & Ron 2010, Hu & Liu 2011). As a chaperone protein, HSP60 plays a wide range of functions in the mitochondrion, including the folding of newly synthesized proteins, the appropriate translocation and folding of proteins within organelles, and the refolding of aggregating or misfolded proteins (Hartl & Hayer-Hartl 2002, Tatsuta 2009, Hu & Liu 2011). New evidence has emphasized the link between ER and mitochondria during stress response (Zhao *et al*. 2002, Haynes & Ron 2010, Hu & Liu 2011). Mitochondrial stress is induced by calcium and ROS/anti-oxidative signaling in response to ER stress (Hu & Liu 2011, Ghemrawi *et al*. 2018). Consistently, our study showed that ER stress directly induced mitochondrial stress protein HSP60 expression (Fig. 2A) and impaired mitochondrial function and membrane potential in the hepatocytes of mice (Fig. 2B and C). On the other hand, overexpression of HSP60 also induced ER stress by increasing GRP78 and CHOP protein levels (Fig. 3A). These results suggested a further functional connection between ER and mitochondria in response to stress.

Interestingly, the knockdown of HSP60 inhibited GRP78 protein expression greatly but had no effect on CHOP expression in the hepatocytes of mice (Fig. 4E). Unfolded or misfolded proteins are recognized by GRP78, which is accompanied by the initiation of ER stress, which ultimately leads to increased CHOP expression and apoptosis (Ghemrawi *et al*. 2018). It is possible that HSP60 may be involved in the initiation regulation of ER stress, but not in the ER stress-induced apoptosis signaling pathway. Therefore, the underlying mechanism requires further investigation.

A number of recent reports have shown that mitochondrial dysfunction plays an important role in insulin resistance (Perez-Carreras *et al*. 2003, Pessayre & Fromenty 2005, Mitchell *et al*. 2009). Consistent with these results, we found that increased mitochondrial stress protein HSP60 inhibited the insulin signaling pathway and impaired insulin sensitivity (Fig. 3B), demonstrating that HSP60 may have a negative effect on hepatic insulin sensitivity. Accumulation of HSP60 can induce insulin resistance in skeletal muscle cells by stimulating the release of pro-inflammatory cytokines (Marker *et al*. 2012, Habich & Sell 2015). In addition, high HSP60 serum concentrations were also reported in type 2 diabetic subjects (Dasu *et al*. 2010). These results suggest that stress-induced HSP60 might stimulate inflammation resulting in metabolic dysfunction and insulin resistance, but the underlying mechanism still requires further investigation. Interestingly, it was reported that rosiglitazone enhanced insulin sensitivity and mitochondrial HSP60 level, which resulted in improved glucose tolerance in the adipocytes of ob/ob mice (Wilson-Fritch *et al*. 2004), suggesting that mitochondrial HSP60, in the UPR^mt^ pathway, may have distinct functions in different tissues *in vivo*.

HFD or ER stress has been well-documented to induce SREBP1 activation associated with hepatic lipogenesis and steatosis (Yoshiuchi *et al*. 2009, Yecies *et al*. 2011, Luo *et al*. 2018). Consistent with these findings, we also found that HFD or ER stress can induce SREBP1 signaling activation, accompanied by increased hepatic lipogenesis (Fig. 4A, B, E and F). A recent study showed that mitochondrial stress is linked to lipid homeostasis in *C. elegans* and human cells (Kim *et al*. 2016). Mitochondrial dysfunction is associated with hepatic lipogenesis and steatosis (Perez-Carreras *et al*. 2003, Pessayre & Fromenty 2005, Mitchell *et al*. 2009). Consistent with HFD or ER stress-induced SREBP1 signaling activation, HSP60 overexpression activated mTORC1-SREBP1 signaling and hepatic lipogenesis (Fig. 4C and D), whereas the knockdown of HSP60 suppressed ER stress-induced mTORC1-SREBP1 signaling and hepatic lipogenesis (Fig. 4E and F), suggesting that HSP60 might be involved in ER stress-induced mTORC1-SREBP1 signaling associated with lipogenesis and steatosis (Fig. 4G). In the future, a liver-specific, genetically modified animal model might be necessary to clarify the role of HSP60 in lipid metabolism *in vivo*. In addition, whether HSP60 directly induces mTORC1-SREBP1 signaling needs further investigation.

To summarize, we have discovered that ER stress can induce mitochondrial stress, and the mitochondrial stress protein HSP60 is a novel regulator of mTORC1-SREBP1 signaling in control of hepatic lipid metabolism. Our findings indicate that targeting HSP60 might provide a new strategy to counteract HFD- and ER stress-induced hepatic steatosis and insulin resistance.

## Declaration of interest

The authors declare that there is no conflict of interest that could be perceived as prejudicing the impartiality of the research reported.

## Funding

This work was supported by grants from the National Nature Science Foundation of China (91957113, 31871180, and 31471131 to F H; and 81800758 to W M).

## Author contribution statement

T X and W M performed collection, analysis, and assembly of data and prepared the first draft of the manuscript; X L, H L, and F Z performed data collection; F H and W M performed conceptualization and design, data analysis and interpretation, manuscript writing, financial support, and approved the final manuscript. All authors reviewed and approved the manuscript. F H is the guarantor of this work and, as such, had full access to all the data in the study and takes responsibility for the integrity and accuracy of the data.
